# Adapting, Pilot Testing and Evaluating the Kick.it App to Support Smoking Cessation for Smokers with Severe Mental Illness: A Study Protocol

**DOI:** 10.3390/ijerph15020254

**Published:** 2018-02-03

**Authors:** Sharon Lawn, Joseph van Agteren, Sara Zabeen, Sue Bertossa, Christopher Barton, James Stewart

**Affiliations:** 1 Flinders Human Behaviour and Health Research Unit, Department of Psychiatry, Flinders University, Adelaide 5042, Australia; Joseph.VanAgteren@sahmri.com (J.v.A.); sara.zabeen@flinders.edu.au (S.Z.); sue.bertossa@flinders.edu.au (S.B.); 2 Wellbeing and Resilience Centre, South Australian Health and Medical Research Institute, Adelaide 5000, Australia; 3 Social Health Sciences Unit, Flinders University, Adelaide 5042, Australia; Chris.barton@monash.edu.au; 4 Kick.it Operations Pty Ltd., Adelaide 5000, Australia; james@kick.it

**Keywords:** smoking cessation, mental illness, youth, co-design, qualitative research, technology, protocol

## Abstract

(1) Background: While the prevalence of tobacco smoking in the general population has declined, it remains exceptionally high for smokers with severe mental illness (SMI), despite significant public health measures. This project aims to adapt, pilot test and evaluate a novel e-health smoking cessation intervention to assist relapse prevention and encourage sustained smoking cessation for young adults (aged 18–29 years) with SMI. (2) Methods: Using co-design principles, the researchers will adapt the Kick.it smartphone App in collaboration with a small sample of current and ex-smokers with SMI. In-depth interviews with smokers with SMI who have attempted to quit in the past 12 months and ex-smokers (i.e., those having not smoked in the past seven days) will explore their perceptions of smoking cessation support options that have been of value to them. Focus group participants will then give their feedback on the existing Kick.it App and any adaptations needed. The adapted App will then be pilot-tested with a small sample of young adult smokers with SMI interested in attempting to cut down or quit smoking, measuring utility, feasibility, acceptability, and preliminary outcomes in supporting their quit efforts. (3) Conclusions: This pilot work will inform a larger definitive trial. Dependent on recruitment success, the project may extend to also include smokers with SMI who are aged 30 years or more.

## 1. Introduction

Tobacco smoking is the leading cause of preventable disease globally. Nearly six million people die from tobacco related disease worldwide each year, and $157 billion in health-related economic losses are directly attributable to smoking [1]. It remains the key modifiable risk factor for development of a number of chronic diseases, including cardiovascular disease, and accounts for 8% of total burden of disease [2]. Almost 3 million Australians still smoke cigarettes, and 1.8 million, or two out of every three smokers, will die from diseases attributable to their smoking [3]. Yet, smoking cessation delivers significant health benefits. Mortality from smoking-related causes diminishes progressively with increasing time since stopping smoking and does not differ significantly between smokers and people who have never smoked where smokers stop prior to age 45 [3,4]. Other benefits of cessation include monetary savings, potentially alleviating financial pressure on those with limited income [5], improved mental health [6], and reduced stress [7].

Smokers with mental illness experience greater tobacco-related disease. While the prevalence of tobacco smoking in the general adult population in Australia has dropped to under 16% [8], it remains exceptionally high and has not changed in over 20 years for smokers with mental illness. For example, data from the large-scale Australian Study of High Impact Psychosis shows that smoking prevalence has remained the same from 1998 (65%) to 2010 (67%) [9,10]. Almost half of all cigarettes smoked in the U.S., the UK and Australia are by people with mental illness [11,12,13], and up to 88% of people with schizophrenia smoke [14]. Life expectancy for people with severe mental illness is 25 years shorter than the general population, and smoking is argued to be the main contributor to this early mortality in this population [15]. These statistics stress the importance of developing interventions that assist all those with mental illness who smokers to quit smoking. In Australia, the National Tobacco Strategy 2012–2018 and the SA Tobacco Control Strategy 2017–2020 both stress the priority to address high smoking rates in young people and strengthening efforts to reduce smoking among mental health populations [16,17].

Addressing smoking early is important because quitting in adulthood is less likely among those who had childhood and adolescent mental health problems [18,19]. A study with 183 adolescent psychiatric inpatients (aged 13–17 years) found that 58.5% were regular smokers [20]. In outpatient mental health services, approximately 20% of young clients (aged 12–17 years) smoke [21]; this is likely an underestimate, given that they may avoid or be slow to engage with health services. Young people with severe mental illness (SMI) who smoke warrant special quitting help because their risk of tobacco dependence by adulthood is even higher than for those without mental illness who begin to smoke during adolescence and early adulthood [22,23,24,25,26]. They are more likely to start smoking, progress to daily smoking, and smoke more heavily [27]. Smoking and mental illness symptoms are strongly positively associated, though the direction and nature of their links are understood to involve a complex causality, also involving confounding and bi-directional factors [18]. In their comprehensive review of the evidence related to tobacco use and cessation in people with anxiety, depression or schizophrenia, Ziedonis et al. confirmed this complexity [18]. For depression, for example, causation between smoking and depressive symptoms appears to operate in both directions [28,29]. 

Generally, quitting without assistance can result in up to 5% successful sustained abstinence at 12 months follow-up [30]. Even brief advice from a health professional can double that rate [31], and behavioural counselling together with pharmacotherapy can increase cessation to up to 35% [32]. Counselling can be delivered through individual face-to-face counselling sessions, group counselling, online programs, or telephone Quitlines, all with Cochrane-level evidence of effectiveness. However, smokers with mental illness have lower smoking cessation rates, suggesting that population-wide programs do not work equally in all and therefore need to be tailored to these smokers [18]. Systematic reviews and studies on adolescents and young adults using behavioural interventions show a need for new, more inclusively designed interventions [33,34]. Like their peers, young adults with mental illness (YAMI) are increasingly ‘digital natives’ [35,36]. Preliminary work in the U.S. confirms that they engage well with e-health interventions [37,38].

The existing evidence for adult smokers with mental illness, generally, confirms that they would like to quit smoking, and that they make as many quit attempts as general population smokers [39,40,41,42,43,44]. However, they may have greater difficulty converting quit attempts into sustained abstinence, even with cessation support [45,46]. They tend to relapse more often and earlier than other smokers, even when provided with current best evidence treatments [43,47]. Indeed, having a mental illness at the time of stopping smoking is a risk factor for relapse to smoking, even after one year of abstinence [47,48]. Barriers to adult smokers with mental illness sustaining an attempt to quit smoking include heavier nicotine dependence, more cigarettes smoked per day, lower self-efficacy, lower use of evidence-based cessation aids, pro- smoking social contexts and networks, and stress [49,50]. In many services in contact with adolescents and adults with SMI (mental health, substance use disorder treatment centres and homeless shelters), smoking is institutionalized [51,52,53]. Adult smokers with mental illness report lack of encouragement to quit, particularly by health professionals [39,42,44]. Health professionals report lack of knowledge and lack of confidence to address it with their clients [54,55]. They hold several misconceptions that: smoking is a necessary self-medication; quitting interferes with clients’ recovery; smoking is the lowest-priority concern for people with acute symptoms; clients are not interested in quitting; and they are unable to quit smoking [39,49]. 

Despite growing interest in e-mental health preventative/treatment interventions, little supporting literature exists to guide their design, development and evaluation of effectiveness, particularly for mental health populations where use and interest is largely unknown. Recent research has investigated acceptability, feasibility and use of technology by youth with mental illness (aged 15–25 years), mental health service staff and community youth-focused organizations in a rural context [56]. A recent review also examined behaviour change models for informing digital technology interventions for people with mental illness [57]. That review confirmed that rates of smartphone ownership among people with mental illness (including schizophrenia and bipolar disorder) are comparable with the general population. It also highlighted the effectiveness of smartphone Apps for assisting specific mental health populations with managing their mental health. A recent U.S. study involving 176 participants with symptoms of depression or anxiety in the field-testing of a suite of mental health support Apps found that many participants had health Apps on their smartphone and used them at least once per day. It also found that tracking and habit building features were the most popular purpose for using these Apps [58]. 

The current research will apply what has been learned from the above research to investigate, develop and test the use of the Kick.it App to support smoking cessation with YAMI (aged 18–29 years). This work can inform further pilot work and a larger trial of the Kick.it App for people with severe mental illness. It may also inform the development of future App technology to support health behaviour change, generally, with this population.

## 2. Materials and Methods

The aim of this project is to adapt, pilot-test and evaluate a novel e-health smoking cessation intervention to assist relapse prevention and encourage sustained smoking cessation for YAMI. Action research will underpin the adaptation of the Kick.it App to the target population, using a participatory and collaborative approach to maximize consumer involvement and applicability to their needs and preferences [59]. This approach will allow stakeholders from both the research team and participants who are YAMI to be involved through iterative and active cycles of designing, reflecting and observing, to support the App’s design for this population. This iterative process will foster joint ownership of project outcomes and allow us to build on learning over time.

### 2.1. About the Kick.it App

Kick.it is an interactive smoking cessation smartphone application (App) that is developed using the Intervention Mapping (IM) framework [60]; a rigorous theory-driven intervention development approach. Formative research using interviews, a focus-group, a comprehensive literature review and stakeholder input, was used to guide a needs analysis to determine the target focus of the App. This needs analysis resulted in a logic model, highlighting the theoretical determinants that needed to be addressed by the App in order to ensure successful smoking cessation. These determinants, stemming from the Theoretical Domains Framework (TDF) [61] were further dissected into change objectives, which were matched with evidence-based theoretical behavior change techniques (BCTs) [62]. The chosen BCTs were subsequently translated into functional components as well as specific content that needed to be used. As the work on BCTs largely stems from consolidation of psychological theory that pre-dates the eHealth and mHealth era, and therefore does not specify effective ways of designing technology to achieve behavior change, the team used the Persuasive Systems Design (PSD) framework [63] to further optimise functional development of App components. An in-depth description of the development process for the Kick.it App is published elsewhere [64].

The resulting end-product is an App aimed at optimizing engagement and adherence. It centers on a social network which, in addition to serving the purpose of allowing provision of social support [65], helps attract users to and retain engagement with the App [66]. It is an easily accessible independent social network where users can get support from other smokers on the same journey or who have made it and are now supporters, as long as they are interested: the App allows for public and private profiles, for those users who wish to quit on their own. The App allows for non- or ex-smokers to join as well and stimulates users to invite family and other loved ones to provide support by joining the App. The App provides content aimed at educating these supporters on how to optimally support smokers who want to kick the habit, e.g., by recording an audio message aimed at increasing motivation. 

Using principles from ecological momentary assessment [67], App users are prompted to record their smokes and craves, their mood-state and their trigger-behaviour, thereby forming a detailed profile of their own smoking and quitting habits. Stored information then provides instant feedback on their progress. Immediately after logging craves, users are presented with evidence-based education (e.g., pharmacological support videos) and exercises using the Kick stack, which presents random content in various formats aimed at helping users get through cravings. It delivers an endless and always different stack of cards with entertaining Graphics Interchange Formats (GIFs—animated short videos) to prompt ideas for actions and strategies to get through each craving. It also delivers curated entertaining, motivational and educational videos from YouTube, games and articles to promote engagement and sustain interest. 

This combined feature, logging smokes and craves when they appear, and providing different content at a random interval, serves an arguably equally important purpose of driving users back to the app on a consistent basis. This approach is based on the Hook Model [68] where habit-forming Apps and games target variable reward. Users need a trigger (internal or external); this leads to an action (opening the App); they then search for the variable reward as dopamine is released in anticipation (scrolling the newsfeed, finding a comment, seeing a photo tagged with them in it, swiping though the Kick Stack, etc.); they find the reward and get a small endorphin hit and then do some work to prime the trigger for next time (for example, comment on a post, upload a photo).

Currently, the Kick.it App is being developed for the general population, to be freely available, and is almost at the minimum viable product stage of testing for the iPhone, with a small number of users participating in alpha-testing. The App is expected to be tested on the first set of test subjects via a formal evaluation early to mid-2018. The App has gained support by winning the Hospital Research Foundation 50th Anniversary Award and receiving attention from Apple who have offered to support Kick.it to design and implement integration of their new CareKit technology. Kick.it has capacity to collect an extensive range of analytics data to measure utility and will be integrated with Apple’s ResearchKit software to capture this data and make it available for analysis for this initial trial. Long-term, Kick.it aims to integrate with existing smoking cessation support options such as Quitlines, general practitioners (GPs) and smoking cessation counsellors in providing a professional support platform that uses the Kick.it app to be able to monitor the quit smoking status, and provide a method for early intervention when, for instance, relapse risk is high (based on the logged data by a smoker).

### 2.2. Participants

Participants for this study will be drawn from an Australian metropolitan community. Inclusion criteria will be as follows: 18–29 years; current or former smoker; self-reported current individual or comorbid mental illness (for example—schizophrenia, schizoaffective disorder, bipolar disorder, major depression, post-traumatic stress disorder, generalized anxiety disorder); currently in receipt of case management support from public community mental health services or has made contact with the government-funded Quitline (free phone-based smoking cessation support and information service to the South Australian community); have attempted to quit smoking in the past 12 months; and able to provide informed consent as determined by the person’s mental health care coordinator or doctor. Exclusion criteria will be as follows: active suicidality or active psychosis as endorsed by the person and their mental health care coordinator or doctor; has never smoked; does not own or have access to a smartphone; sensory or motor impairment that would prevent completion of study procedures; unable to provide informed consent (for example, due to low IQ or severe cognitive impairment).

### 2.3. Recruitment

Potential participants will be identified and their voluntary participation flagged by South Australian Quitline staff or their community mental health service care coordinators. Contact to express their interest in participating in the study will be done by the young person contacting the lead researcher directly via phone or email, or with support from their mental health care coordinator or from Quitline staff who will provide their contact details to the lead researcher for follow-up, with the young person’s consent. Self-reported smoking status will be determined using the following questions “Which of the following best describes your smoking status?” and respond by selecting from “I’m a smoker, I smoke daily”, “I’m an ex-smoker, I never smoke now”. This widely used item to elicit smoking history has demonstrated accuracy in past research [69]. Given the limited research with this population age group, we will add a further question: “I’m a smoker; I smoke on at least 2 days per week”. Participants can elect to participate in individual cycles or all cycles of the data collection process, and will be free to withdraw at any time without adverse impact on their standing with their mental health service or the Quitline. 

### 2.4. Data Collection

**Figure 1:**
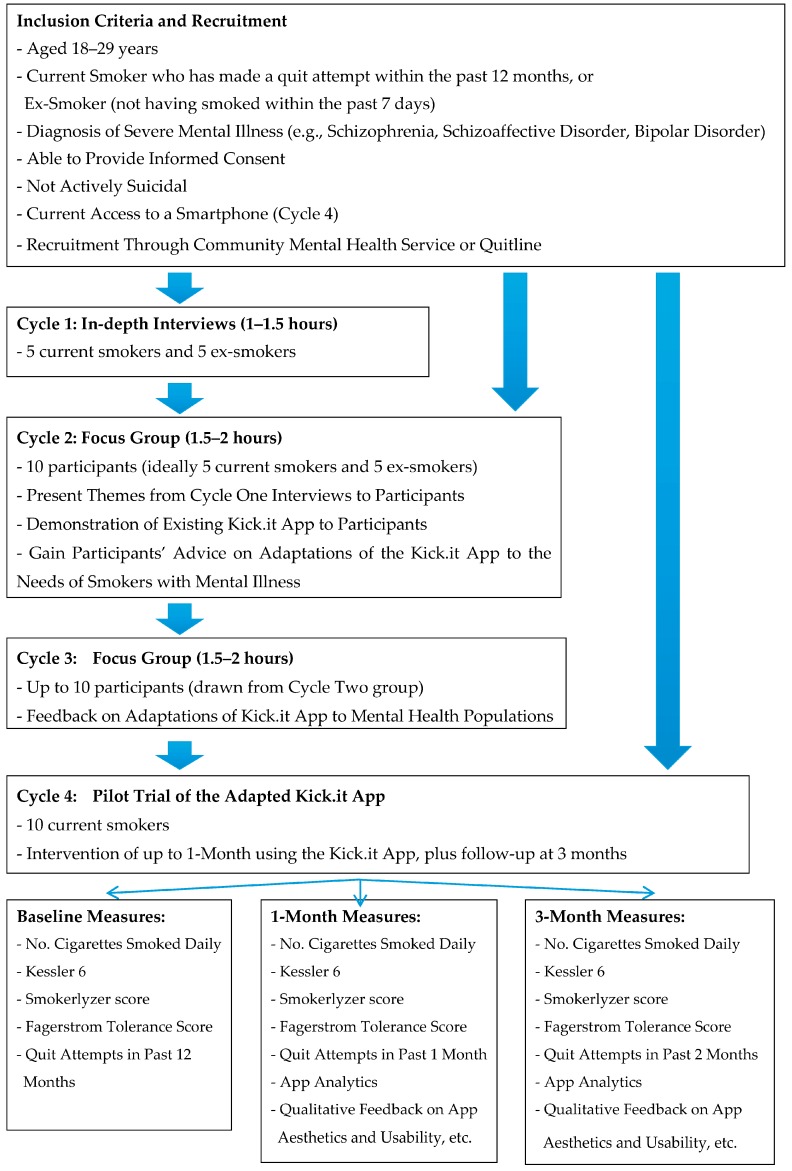
Outline of Kick.it trial protocol components.

To achieve the study’s aim, the research team will undertake four cycles of data collection using the action research approach where each cycle informs the next stage of the research. Sample size for each cycle has been determined by the research team according to the resources available to conduct this pilot work, and also drawing advice from the design of other studies [57]. Where any participants are lost to follow-up, we will attempt to recruit further participants. The four cycles are outlined below and in Figure 1.

Cycle 1: Individual interviews with five YAMI (smokers) who have attempted to quit smoking and five YAMI (ex-smokers). An interview guide will be developed that aligns with the domains of interest relevant to quitting for this population, as determined by the existing evidence, and also the broad domains upon which the Kick.it App has been developed. This will include: available supports, coping strategies, communication and help-seeking styles, motivation, managing dependence, habits and environmental influences [70]. Interviews will explore what meaning they derive from smoking, types of support participants found helpful, what motivators were most applicable to their needs, barriers and facilitators to using smoking cessation supports and aides, how they managed their mental health while attempting to quit, involvement of others in their quit attempts, and what strategies ex-smokers used to help stay quit [51]. Specific interview questions will include the following examples: starting to smoke (e.g., “Describe to me what it was like when you first started smoking”), life as a smoker (e.g., “What place does smoking have in your life?”), and the quitting process (e.g., “When you started the intervention, what kinds of things were you looking for help with?”) [71]. 

Cycle 2: A focus group workshop comprising 10 YAMI (smokers and ex-smokers) will review the tentative themes that emerge in the first cycle and will have the opportunity to provide any further feedback on the fit with their experience as a YAMI. Participation will be open to participants from the first cycle, should they wish to be involved in this step. During this focus group workshop, participants will be shown the Kick.it App and will be asked to provide their advice on its various features, also given the information obtained from the first cycle data. The workshop will be structured around the various functions of the Kick.it App to enable systematic organization and collection of participants’ feedback.

Cycle 3: Combined data from the first two cycles will inform the adaptation of the Kick.it App by the e-health developers (under supervision of Joseph van Agteren and James Stewart). Once adaptations have been completed, as many participants as possible from the second cycle will be invited to a further focus group to provide final feedback on the adaptations. 

Cycle 4: App testing with a sample of 10 YAMI (smokers) interested in using the App to assist them to quit smoking. Participation will be open to YAMI from the earlier cycles, if they wish to be involved. Each participant will be provided with individual training and support to use the App’s functions. In the first fortnight, the Technical Support Project Officer (Joseph van Agteren) will contact each participant to flag any technical queries they are having in relation to using the App. Participants will also be provided with a generic project contact number for any troubleshooting enquiries they might have during the App testing period. 

To examine acceptability, feasibility, utility and preliminary outcomes of the tailored Kick.it App at 1 and 3-months follow-up, the following measures will be tracked from the App analytics: Continuous abstinence (not smoking since a quit date was set); Quit attempts lasting at least 24-h; Reduction in number of cigarettes smoked by >50%; Uptake of nicotine replacement therapy (NRT); and, Use of the Quitline to augment quit efforts. To support these analytics and for comparative purposes, we will also conduct individual interviews at 1 and 3 months, to collect self-reported measures of the above, in addition to self-reported 7-day point prevalence smoking abstinence [72,73,74], self-rated psychological distress, as measured by the widely used and validated K6 [75], at baseline, 1 and 3 months. To further verify smoking status, we will also collect measures of carbon monoxide (CO) at commencement of using the App, 1 month and 3 months using the Smokerlyzer^®^ (Niche Medical, Perth, Australia) [76] which provides a quick and non-invasive measure the amount of CO on a smoker’s breath and biochemically establishes their smoking status. Qualitative data will also be sought during these interviews, seeking participants’ feedback on the App’s aesthetics and perceived usability, the App’s ability to focus their attention, and their felt involvement and intention to engage in ongoing use of the App. 

For cycle 4, the following measures will guide outcomes data collection:

- Nicotine dependence: measured by a 2-item short form of the Fagerstrom Tolerance [77] Questionnaire. Scores range from 0–6 and are calculated by summing the points for (1) time to first cigarette smoked after waking (in minutes) and (2) number of cigarettes smoked per day.
- Prolonged abstinence: “Since (date of two weeks following baseline survey) did you smoke at all, even part of a cigarette?” [78].
- Quit attempts: commonly used item that asks “How many serious attempts to stop smoking have you made in the last 12 months? By serious attempt I mean you decided that you would try to make sure you never smoked again...” [78].
- Psychological distress: Brief 6-item K6 screening scale with robust psychometric properties [75]; it asks respondents to report how often they feel “nervous”, “hopeless”, “restless or fidgety”, “so depressed that nothing could cheer you up”, “that everything was an effort”, “worthless” over one of two recall periods (past month, and worst month).

We will aim to recruit further participants in the event that any participants in cycle 4 are lost to follow-up. We intend to have regular contact with participants, to monitor their progress and answer any questions or concerns they have about using the App, in order to increase retention. Feedback from participants on the acceptability and use of the above suite of outcome measures will inform the acceptability and feasibility of using these measures, modifying them, or seeking to use other measures for further pilot work and definitive trials.

Ethical approval to conduct this study has been provided by the Southern Adelaide Clinical Human Research Ethics Committee (16:17). All participants will be provided with a participant information sheet and consent form. A small honorarium will be provided to participants at each cycle of the data collection process.

### 2.5. Data Analysis

All interviews and focus groups will be audio-recorded and professionally transcribed to ensure data integrity. Interview data from Cycle 1 will be analysed using Thematic Analysis to report patterns within the data, using Braun and Clarke’s six phase process: familiarising oneself with the data, generating initial codes, searching for themes, reviewing themes, refining and naming themes and producing the report [79]. The first three transcripts will be read and re-read independently by two members of the research team (SL and SB), who will then independently manually open-code them to identify data features that seem interesting and meaningful. Codes will evolve, following critical review and discussions between the researchers. The lead researcher (SL) will then code the remaining interviews based on the research team’s agreement on the preliminary coding structure. Mind maps will be used to assist the researchers to understand relationships, patterns and emerging concepts within the data. Reflexivity and interpretive rigour throughout the data analysis will involve incorporation of researcher memos and reflections [80]. The researchers will then meet to determine a coherent logical story that incorporates extracts from the participants’ own words to exemplify each theme. Data saturation will not be possible with the small sample size of five smokers and five ex-smokers.

Focus group data from Cycle 2 and Cycle 3 will be analyzed using Framework Analysis [81,82], given the pre-existence of known broad domains of interest and the need to apply outcomes of these stages to the adaptation of the Kick.it App. These domains will include the App’s aesthetics and perceived usability, its ability to focus attention, participants’ felt involvement and intention to engage in ongoing use of the App. 

Acceptability, feasibility, utility and preliminary outcomes data collected from self-report measures and activities during Cycle 4 will be analyzed using a range of statistical methods. A number of measures collected from the App analytics will be reported as descriptive statistical data, given the small sample size [83]; for example, uptake of NRT, and use of the Quitline. Repeated Measures ANOVA [84] will be used to analyse all measures that are collected across the three time points of baseline, 1 month and 3 months. This will apply to analysis of data arising from, for example, the Fagerstrom Tolerance Questionnaire, the Brief 6-item K6, number of cigarettes smoked, and CO measures. In the event that participants drop out before the third outcome measure can be collected, resulting in only 2 time points of data being available, paired sample *t*-tests [85] will be used to analyse these measures. For analysing data on prolonged abstinence, where the answer will be either ‘yes’ or ‘no, the Chi-square test [86] will be used.

## 3. Discussion—Strengths and Limitations

The adverse health impacts of smoking for people with mental illness is now clearly established. While there are many Apps available to support people to address lifestyle issues such as smoking, alcohol, diet and exercise, and a growing number of Apps available to assist people to manage symptoms of their mental health conditions and keep track of their medication use, there are few Apps available that are tailored to the needs of people with SMI who have comorbid addiction to tobacco. A strength of the current study is that it aims to elicit the direct experiences of YAMI who smoke, through a series of collaborative cycles of consultation, to inform the adaptation of a smoking cessation App to the needs of people with comorbid mental illness.

Another strength of the current study is the use of the Kick.it App. Although still in development, the App’s features are based on sound behaviour change theory [57,83]. Its features also align with many elements suggested as important within the recently published guidelines for App development [87,88]. These include features that enable longitudinal data collection, realtime behaviour monitoring and feedback, and the ability to tailor feedback to the App users’ changing needs over time [57].

Few pilot studies that investigate the use and engagement with lifestyle Apps collect data beyond a few days [57,83]. A further strength of the current study is the intention to follow-up with participants using the App for up to 3 months, and to collect a broad range of measure of ulility, feasibility and acceptability from participants using App analytics, self-report and CO verification at commencement, 1 and 3 months using the Smokerlyzer^®^ to biochemically establish their smoking status [76]. However, this time period is also a limitation, given the need for a more longitudinal trial with a larger sample to better understand the mechanisms that would increase App users’ longer-term engagement with the App, and the App’s impact on sustained smoking cessation. Further pilot work with a larger sample, and a larger trial that includes a control group, would allow us to minimise any bias within the sample, according to diagnosis, by matching smokers and ex-smokers based on diagnosis, given the potential for them to have differing needs in relation to quitting, and management of their comorbid mental health conditions [18,51]. This pilot work and its goals are more focused on eliciting preliminary utility, feasibility and acceptability data to inform further investigations. The sample is simply too small to gain any meaningful data from such a matching process. Other demographic variables of interest such as age and gender could also be explored within a larger study. A larger study could also compare the performance of the Kick.it App with established smoking cessation Apps, such as the Australian Government funded MyQuitBuddy which is widely supported by the network of Australian Quitlines and which has reported a smoking cessation success rate of 37% [89].

Currently, the Kick.it App only targets tobacco smoking. The App is designed to be rolled out to other addictions, but that is beyond the scope of the current study. Also, whilst there is growing interest in the potential benefits of e-cigarettes for smokers with mental illness, no e-cigarette specific support is provided within the Kick.it App at present, considering the current status of Australian legislation which bans the sale of e-cigarettes within Australia. When the App is made available to the wider public outside of Australia, this topic will be revisited. 

Another potential limitation related to the small sample size, is that the likely nuances and complexities of providing effective smoking cessation support to people with SMI will not be fully accounted for in participants’ feedback during the App adaptation cycles. A recent large U.S. trial of general population smokers found that they preferred gain-framed features rather than loss-framed features; that is, they preferred features that told them how their health was improving rather than being given information about the harms of smoking [90]. However, it is unknown whether YAMI will have similar preferences.

It is also unclear whether and to what extent drop-out will be an issue in the current study. As stated earlier, we will endeavour to recruit further participants to the 4th Cycle, and are guided by a recent review of App use for health behaviour change interventions that found, of 17 studies, 10 achieved a high retention rate (>80%) in the intervention group [83]. That is, we expect that there will be some drop-out, and will determine further data collection and analysis based on this likelihood.

A further issue of concern that will require attention during the current study is how participants are supported in the event that they endorse a significant amount of psychological distress on the K6, or it becomes evident that their mental health deteriorates during the study, either as a result of their attempt to quit smoking, or for some other reason. If this occurs, we will ask the participant if they wish to proceed and also suggest that they make contact with their dedicated case manager at the outpatient mental health service, or their local general practitioner. Given the relatively short timeframe for the 4th cycle (3 months), we expect that participants will be continuous clients of the mental health service for the duration of the study, therefore minimising this concern.

Despite the limitations outlined above, we agree with Zhao et al.’s emphasis, that, “Apps that offer even a small health benefit could still be a valuable public health intervention” (p. e294) [83]. This is particularly important for smokers with SMI of all ages for whom public health smoking cessation measures have had little impact. 

## 4. Conclusions

This project commenced in 2017 and will conclude in 2018. This pilot work will inform a larger more rigorous and adequately powered trial with this population. Dependent on recruitment success, the project may extend to also include any adult smokers with mental illness given familiarity with smartphone use is increasingly common across the broader population. Further directions for research would also be to target an even younger population (i.e., adolescents) in future studies using the App, and to investigate how mental health professionals can incorporate the use of this and other health focused Apps into their clinical care. There is also a need to understand the mechanisms that would increase App users’ longer-term engagement with Apps, and address potential barriers through improved App design and support strategies, to effect sustainable behaviour change.
